# Terahertz spectra of proteinuria and non-proteinuria

**DOI:** 10.3389/fbioe.2023.1119694

**Published:** 2023-02-17

**Authors:** Zhenrui Xue, Ping Mao, Ping Peng, Shihan Yan, Ziyi Zang, Chunyan Yao

**Affiliations:** ^1^ Department of Transfusion Medicine, Southwest Hospital, Third Military Medical University (Army Medical University), Chongqing, China; ^2^ Department of Clinical Laboratory, Sichuan Provincial Crops Hospital of Chinese People’s Armed Police Forces, Leshan, Sichuan, China; ^3^ Chongqing Institute of Green and Intelligent Technology, Chinese Academy of Sciences, Chongqing, China; ^4^ Chongqing School, University of Chinese Academy of Sciences (UCAS Chongqing), Chongqing, China

**Keywords:** proteinuria, terahertz time-domain spectroscopy, protein, absorption coefficient, pH

## Abstract

In clinical practice, proteinuria detection is of great significance in the diagnosis of kidney diseases. Dipstick analysis is used in most outpatient settings to semi-quantitatively measure the urine protein concentration. However, this method has limitations for protein detection, and alkaline urine or hematuria will cause false positive results. Recently, terahertz time-domain spectroscopy (THz-TDS) with strong hydrogen bonding sensitivity has been proven to be able to distinguish different types of biological solutions, which means that protein molecules in urine may have different THz spectral characteristics. In this study, we performed a preliminary clinical study investigating the terahertz spectra of 20 fresh urine samples (non-proteinuria and proteinuria). The results showed that the concentration of urine protein was positively correlated with the absorption of THz spectra at 0.5–1.2 THz. At 1.0 THz, the pH values (6, 7, 8, and 9) had no significant effect on the THz absorption spectra of urine proteins. The terahertz absorption of proteins with a high molecular weight (albumin) was greater than that of proteins with a low molecular weight (β2-microglobulin) at the same concentration. Overall, THz-TDS spectroscopy for the qualitative detection of proteinuria is not affected by pH and has the potential to discriminate between albumin and β2-microglobulin in urine.

## Introduction

Protein analysis of urine is an important clinical test, mainly used for diagnosis and treatment monitoring of kidney diseases, such as nephritis and renal failure. Healthy people lose less than 150 mg of protein from urine daily; therefore, a total protein excretion rate higher than 150 mg/24 h is considered proteinuria (Lamb et al., 2009). Proteinuria can be divided into glomerular proteinuria and tubular proteinuria according to the protein type. Glomerular proteinuria is dominated by albumin, whereas tubular proteinuria is dominated by β2-microglobulin (Tampe et al., 2021). At present, the protein qualitative test of urine is commonly performed using the sulfosalicylic acid method, the heated acetic acid method, and the dipstick test (Kumar and Gill, 2018). The sulfosalicylic acid method reacts with both albumin and globulin with high sensitivity (50 mg/L) and is preferred for screening tests, but strongly alkaline urine (pH > 9) or strong acid urine (pH < 3) is prone to false negative. A reaction time of more than 1 min will increase the probability of positive results, so the results need to be observed on time (Ridley, 2018). The heated acetic acid method reacts to both albumin and globulin but is less sensitive (150 mg/L) (Rahayu and Rustiana, 2020). The dipstick test is sensitive to albumin but not to globulin, and its sensitivity (70 mg/L) is slightly lower than that of the sulfosalicylic acid method. False positives can occur when urine pH is elevated due to renal tubular acidosis, therapy of alkaline drugs, diet (vegetarian) (Carroll and Temte, 2000; Roberts, 2007; Clarkson et al., 2011). However, the dipstick test is often used to screen a large number of samples and medical emergencies duo to its simple operation, low cost. These biochemical methods require additional reagents and often rely on the naked eye to determine the results, which increases the complexity of the operation and reduces the accuracy of the results. It is necessary to develop a rapid and accurate screening method for proteinuria.

Terahertz (THz) waves are electromagnetic waves with frequencies between 0.1 and 10.0 THz, and their energy level range covers the energy levels of low-frequency motions (vibrations and rotations) of many organic macromolecules and hydrogen bonding networks in solution (9). These motions can be identified by fingerprint features in the THz transmission or absorption spectra, absorption changes, and phase changes (Zheng et al., 2014; Yan et al., 2016). Changes of biomolecules, such as change of formation, content, and state, strongly affect the THz spectral characteristics (Niehues et al., 2011; Bye et al., 2014; Klokkou et al., 2022). Recently, THz wave has been proved to have strong interactions with many biochemical molecules such as amino acids, proteins, and deoxyribonucleic acid (DNA) (Esser et al., 2018; Wu et al., 2020; Mancini et al., 2022). Moreover, THz waves have low photon energies (1 THz = 4.1 meV), which is about one million times weaker than the energy of X-ray photons, so it does not cause any harmful ionization in biological molecules (Sirkeli et al., 2018). Because of these properties, THz spectroscopy has emerged as a powerful tool for studying solvated biomolecules (Xie et al., 2014).

Blood is rich in solvated molecules, and many studies have been conducted using THz-TDS spectroscopy to detect blood components. Reid et al. found that there were significant differences in both the absorption spectra and refractive index spectra of the whole blood and thrombus, demonstrating the potential of THz spectroscopy in distinguishing different combinations of substances in human blood (Reid et al., 2013). Furthermore, Torii et al. measured the reflectance of sub-THz radiation on the concentration of glucose and albumin, and concluded that sub-THz radiation can be used to measure blood glucose and albumin (Torii et al., 2017). In addition, Chen et al. found a linear relationship between the THz absorption coefficient and blood glucose level through a quantitative analysis of 70 patients (Chen et al., 2018). Since both blood and urine are liquid biological specimens with complex compositions, and urine is essentially a product of ultra-filtered plasma, the study of THz-TDS spectroscopy for blood composition provides a theoretical basis for the use of THz-TDS spectroscopy for urine protein measurement. Therefore, we hypothesize that THz-TDS spectroscopy has the potential to detect proteins in urine samples and solve the problem of false positives and inaccuracy of the dipstick test in proteinuria screening.

In this study, we propose a new approach using THz-TDS for the qualitative detection of proteinuria. First, we compared the absorption spectra of proteinuria and non-proteinuria. Next, we investigated the effect of pH (6.0–9.0) on the analysis of proteinuria. Finally, we investigated the absorption spectra of β2-microglobulin and albumin to verify the ability of THz-TDS to distinguish different kinds of proteinuria.

## Methods and materials

### Experimental materials

Twenty urine samples (mean age 56 years; range 52–58 years) were provided by the department of laboratory medicine, Southwest Hospital, Chongqing. Our study was approved by Ethics Committee of the First Affiliated Hospital of Army Medical University (No.KY2020227). All the chemicals were purchased from Aladdin (Shanghai, China). Solutions were prepared with deionized water (Millipore, United States). Human Serum Albumin (HSA) (CAS No. 70024-90-7) was purchased from Solarbio (Beijing, China). Urine sample from tubular proteinuria patients which containing human β2-microglobulin was purchased from Prospec-Tany Technogene Ltd (Ness-Ziona, Israel). Urinalysis strips (H12-MA) were purchased from DiRui (Changchun, China).

For proteinuria testing, 20 samples with different protein concentrations and other parameters (pH, leukocytes, bilirubin, *etc.*) were chosen to be similar. The 20 samples were equally divided into four groups based on protein concentration:—(<0.1 g/L), + (0.3–1.0 g/L), ++ (1.0–3.0 g/L), +++ (3.0–6.0 g/L). For the test of effect of pH value on urine samples, HCl and NaOH were used to adjust the pH value of urine samples to 6.0, 7.0, 8.0, and 9.0, respectively. Normal urine sample was selected and divided into two parts, different concentrations of protein (albumin and β2-microglobulin) from 1 g/L to 10 g/L were added to observe the changes in the absorption spectrum of THz.

### Urine test strip analysis

The pH and qualitative protein results contained in the urinalysis strips were reported by the fully automated UC-3500 (Sysmex, Kobe, Japan). Data are presented in the reports on an ordinal scale (as “normal,” “negative,” “positive,” or “nominal concentrations”).

### Experimental equipment and sample cell

**FIGURE 1:**
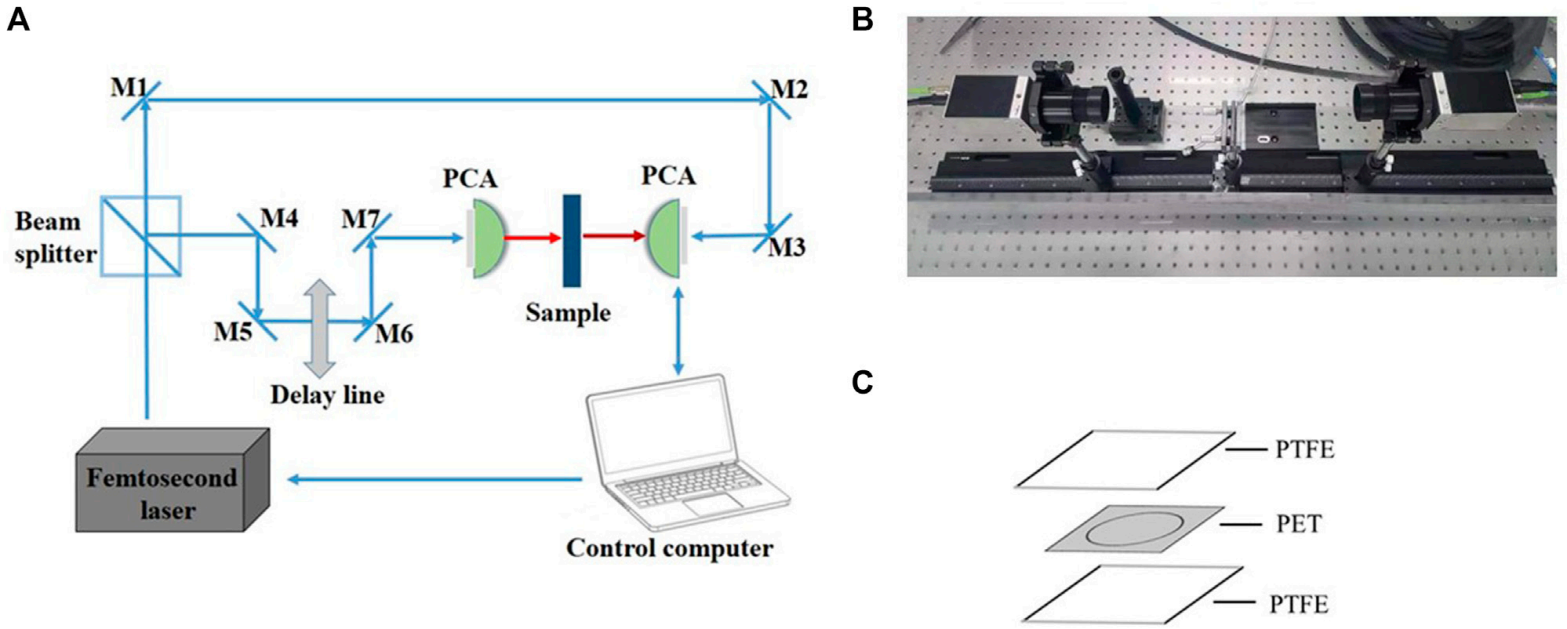
Experimental equipment **(A)** Schematics of the THz-TDS **(B)** The actual experimental setup **(C)** Schematic diagram of the sample cell.

A Picometrix T-ray 5000 fiber-coupling spectrometer (Advanced Photonix, Inc., MI, United States) was used in the experiment (Figures 1A, B). The spectrometer generated and coherently detected the electric field of ultrashort THz electromagnetic pulses in the time domain using femtosecond near-infrared laser pulses and LT-InGaAs photoconductive antenna chips. The femtosecond pulse was produced by a Sapphire oscillator with a repetition rate of 100 MHz, a central wavelength of 1064 nm, and a duration of <100 fs. This pulse was split into two parts by a polarising beam splitter, one as a probe beam shining directly on the photoconductive antenna (PCA) and the other as pump light collected on the other PCA. The detection light was discretely sampled from the terahertz signal irradiating the second PCA to obtain a time domain waveform, which was transformed into the frequency domain using the Fast Fourier Transform. The sampling interval of the THz-TDS was 0.1 ps, and the spectral resolution was 12.5 GHz. More detailed descriptions can be found in our previous reports (Yan et al., 2016; Zang et al., 2019).

For the liquid measurement, the sample cells were prepared as follows: polyethylene glycol terephthalate (PET) double-sided tape (3M, MN, United States of America) of 0.10 mm thickness was cut into square spacers with side dimensions of 22.0 mm, in which a circle with a diameter of 15.0 mm was removed simultaneously, for accommodating the liquid sample (Figure 1C). By sealing the spacer with two polytetrafluoroethylene (PTFE) cover slips (Fisher Scientific, MA, United States) pasted together after the addition of the liquid samples, removal of excessive solution from any side of the spacer was possible to ensure that the spacer was completely filled. With a diameter of 15.0 mm and a thickness of 0.10 mm, the volume of the secure seal spacer was calculated to be ∼17.7 μL, which was also the amount of sample added. The entire procedure takes less than a minute and the materials are inexpensive.

### THz-TDS measurement

The THz frequency range was chosen between 0.5 and 1.2 THz to obtain the maximum signal-to-noise ratio and the most stable signal. Spectral frequency resolution of the spectrometer is 12.5 GHz. The measurement temperature was controlled at 21°C ± 0.4°C. The relative humidity was maintained at < 2.0% by nitrogen gas purging. An empty spacer was used as a reference to eliminate the background effects and each sample was measured seven times. After the sample chamber was prepared, it was sandwiched between a PTFE gasket and a neoprene gasket for measurement, and the frequency spectrum of the measured signal was obtained by using the Fourier transform.

### Data analysis

The solution absorption coefficient is the energy loss of the terahertz beam in the medium. Taking into account the effect of reflection between the PTFE window and the sample, the absorption coefficient can be calculated by the improved Beer-Lambert theoretical formula as follows (Zang et al., 2019).

$$\alpha \left(\omega \right)=\frac{2}{d}\ln\left\{\frac{4n\left(\omega \right)n_{q}\left(\omega \right)}{{\left[n\left(\omega \right)+n_{q}\left(\omega \right)\right]}^{2}}.\frac{{\left[n_{q}\left(\omega \right)+1\right]}^{2}}{4n_{q}\left(\omega \right)}.\frac{1}{A\left(\omega \right)}\right\}$$ (1)

Where *d* is the thickness of the urine sample cell; *n*_*q*_(ω) = 1.43 is the refractive index of PTFE; *A(ω)* is the amplitude ratio of the Fourier transform of the urine sample cell (*I*_*s*_) and the blank sample cell (*I*_*ref*_); *n(ω)* is the refractive index of the sample.

### Statistics analysis

The statistical analysis was performed using the SPSS software package, version 16.0 (SPSS, Chicago, IL, United States). The data are presented as means ± standard deviations (SDs), or medians. For comparisons, Student’s t*-*test and the Mann–Whitney test were applied to continuous variables, while the chi-squared test was applied to categorical variables. The results of THz absorption in urine with different pH values were analyzed by one-way ANOVA and postoperative examination. The statistical differences between each group were determined by the least significant difference (LSD) test. *p* values <0.05 were considered significant.

## Results

### Normal urine and proteinuria identification based on THz spectra

**FIGURE 2:**
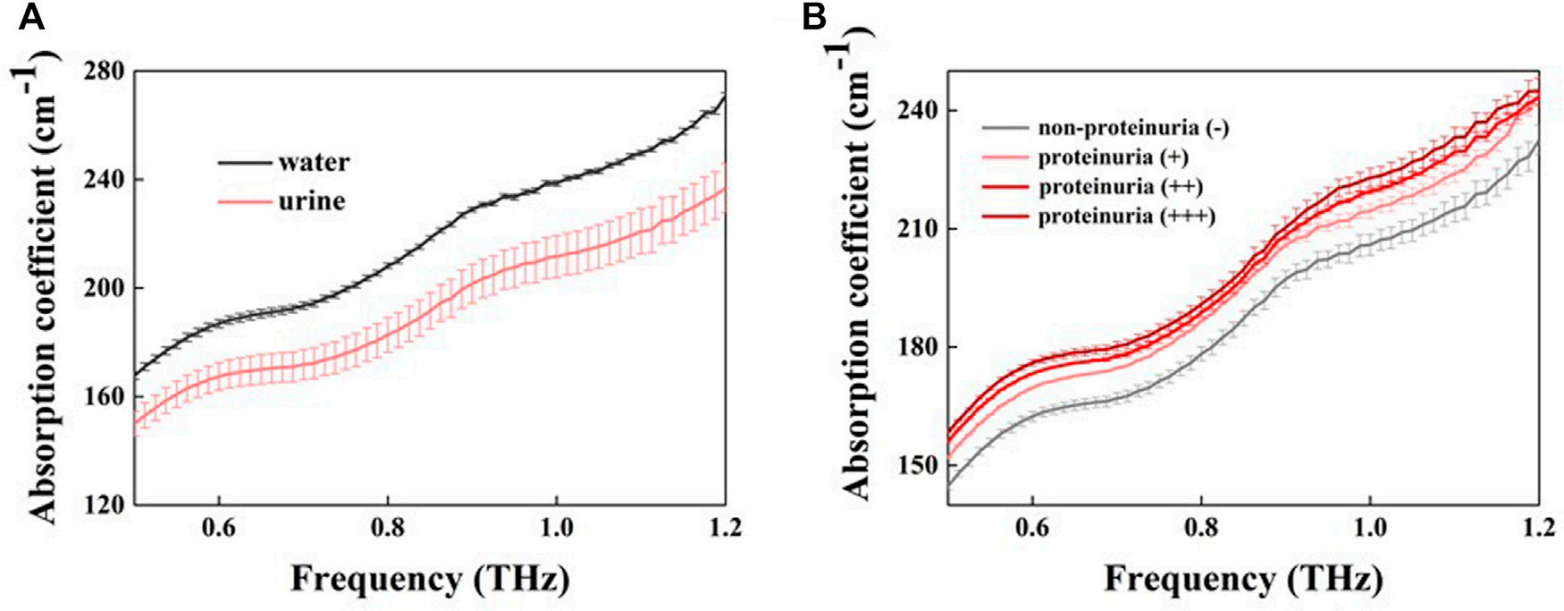
The absorption coefficient of water and urine samples **(A)** Absorption coefficients for pure water (black line) and urine (red line) **(B)** THz absorption coefficients for different concentrations of proteinuria (different red lines) and non-proteinuria (grey lines). Error bars indicate the standard deviation.

All non-smooth curves in the article are Fabry-Perot oscillations produced by thin sample pools and have no effect on the comparison of absorption coefficients between samples. Figure 2 shows the absorption coefficient of urine and pure water in the range of 0.5–1.2 THz. No significant absorption peaks were observed in the THz absorption spectra for the samples due to the disturbance and concealment of the liquid water (Xu et al., 2006). The absorption coefficient of water was higher than that of urine (Figure 2A). At 1.0 THz, the average absorption coefficient of water was 239 cm^-1^, while that of urine was 214 cm^-1^, with a difference of 25 cm^-1^. The largest absorption coefficient difference between the urine samples was 14 cm^−1^ at 1.0 THz. We can observe that the largest absorption coefficient between the urine samples was comparable to the absorption coefficient difference between water and urine, which strongly indicates that THz absorption coefficient is sensitive to some contents in urine. Since the proteinuria samples were normal in other parameters, we therefore inferred protein in urine sample caused the absorption coefficient difference. Figure 2B shows the relationship between absorption coefficient and protein concentration in urinary samples. Proteinuria sample generally have a greater absorption coefficient than non-protein urine sample, moreover, the absorption coefficient increases with increasing protein concentration.

### The effect of pH on urine

**FIGURE 3:**
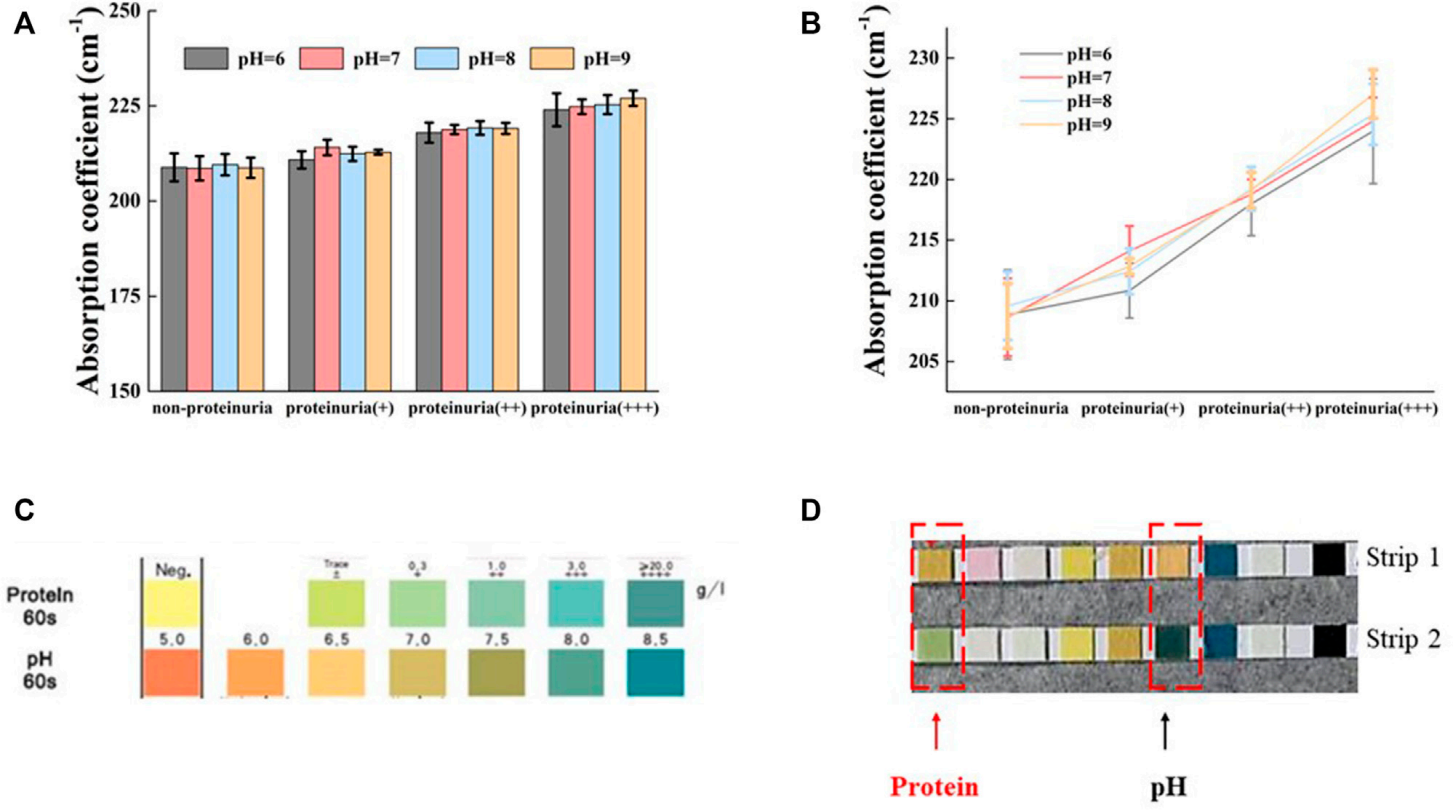
Effect of pH on the absorption of THz in different urine **(A)** Variation of urine absorption coefficient with pH for four different protein concentrations at 1.0 THz **(B)** Absorption coefficients and protein concentrations of urine with different protein concentrations at the same pH **(C)** Colorimetric mapping of urine analysis test strips **(D)** Non-proteinuria (strip 1) appears false positive after raising pH (strip 2). All data are shown as the mean ± standard deviation.

It is well known that alkaline urine (pH > 7.5) can cause false-positive results in the detection of urinary protein by dipstick method (Penders et al., 2002). To confirm whether the detection of protein by THz-TDS method is affected by pH, we used proteinuria samples with different pH value for detection. Here, HCl or NaOH was used to adjust the pH of urine samples to 6, 7, 8, and 9, respectively. The absorption coefficient of proteinuria (different protein concentrations) did not change significantly by different pH value at 1.0 THz (*p* > 0.05, Figure 3A). A linear increase in the absorption coefficient with increasing protein concentration was observed in the absorption coefficient profiles of urine samples with different concentration of proteins at the same pH (Figure 3B). The colorimetric profile of the urine analysis test strips was shown in Figure 3C. The protein concentration of normal urine without pH adjustment was negative with a pH around 6.5 (strip 1, Figure 3D). When this normal urine was adjusted to pH around 8.0, the protein concentration showed + ∼ ++ (strip 2, Figure 3D). The above results demonstrated that the test strip method gave a false positive for protein at high pH, while the THz method was independent of pH with no significant change in absorption coefficient. In other words, the THz-TDS method seemed to be more suitable than the strip method for protein detection in alkaline urine samples.

### The effect of different concentrations and types of protein on urinary absorption

**FIGURE 4:**
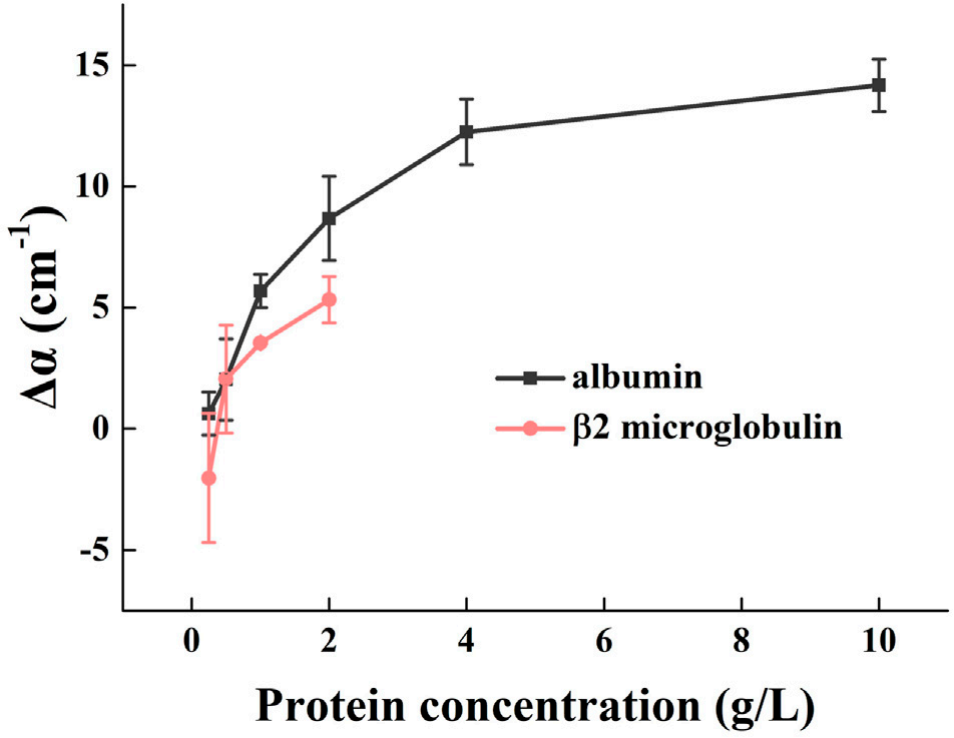
Effect of different types and concentrations of protein on urinary THz absorption. At 1.0 THz, the absorption spectra of albumin and β2 microglobulin at different concentrations. Δα = *α*(c) - α(urine), where α(urine) is absorption coefficient of urine before protein addition.

The absorption spectra of β2-microglobulin and albumin with different concentrations at 1.0 THz are shown in Figure 4. The absorption coefficient of albumin was progressively greater than that of β2-microglobulin with increasing concentration, at concentrations higher than 0.5 g/L (*p* < 0.05). Although the error bars overlap at low concentration (<0.5 g/L), the curves generally show that higher molecular weight protein (albumin) has higher absorption capacity. Albumin is a single polypeptide chain consisting of 585 amino acids, and adopts a heart-shaped 3D structure with three homologous domains I-III; each domain contains two subdomains (He and Carter, 1992). The β2-microglobulin is a single-chain polypeptide composed of 99 amino acids, containing a pair of disulfide bonds within the molecule and no sugars. Comparing these two proteins, albumin has a more complex conformation, which directly affects the dielectric response in the THz range and enhances THz absorption.

## Discussion

In our study, the absorption coefficient increased with increasing protein concentration in the range of 0.5 THz to 1.2 THz, which can be explained exactly by the following theory. We consider the collected urine samples to have the same concentration of all components except for the protein concentration, which can be approximated by defining the samples as different concentrations of protein solutions. When aqueous solutions of various substances were studied, which could not be simply described as a two-component system; a third component was needed. Logically, the component was assumed as a hydrated shell (Penkov et al., 2018). For solvated protein, the total absorption could be decomposed into three components, the volume weighted average of the solute, the solvation water, and the bulk water, defined as follows (Ebbinghaus et al., 2007)

$$\alpha =\left(\alpha_{protein}V_{protein}\right)/V+\left(\alpha_{shell}V_{shell}\right)/V+\alpha_{protein}\left[1-\left(V_{protein}+V_{shell}\right)/V\right]$$ (2)

When protein was added, the solute molecules had a lower frequency mode than the solvent, and the protein solution had a linear decrease in terahertz absorption compared to pure water. In addition, the concentrations of protein in the urine samples collected in this study were all below 0.5 mM, the absorption of solute water molecules in the broader (>10 Å) solute shell layer was enhanced, resulting in a linear increase in the absorption coefficient of the protein solution with concentration (Ebbinghaus et al., 2008).

When we discuss the effect of pH on the terahertz absorption of protein urine, we focused on the analysis of albumin, the main component of urinary protein. Global perturbations of the protein hydration shell caused by pH and local perturbations caused by charge dependent mutations at surface sites can alter the hydration dynamics, producing significant changes in the terahertz absorption spectrum of the protein solution (Ebbinghaus et al., 2008; Born and Havenith, 2009). During the transition from weakly acidic (pH 6.0) to alkaline (pH 9.0), albumin undergoes transitions, including normal type (N) and alkaline type (B) (Qin et al., 2016). The N-B transition undergoes a very subtle change with almost no loss of secondary structure (Leonard et al., 1963), which implies that the protein retains most of its heterogeneity and is chemically identical in the B and N conformations (Babcock and Brancaleon, 2013). Nevertheless, the protein still undergoes de-folding, extending from a tiny spherical structure to a loose chain with no specific spatial structure. This process alters the absorption rate and thickness of the extended hydration shell, which lead to a decrease in absorption (Heyden and Havenith, 2010). However, unfolded proteins have a high density of vibrational modes between 0 and 2.0 THz, leading to an increase in THz absorption (Castro-Camus and Johnston, 2008). The two effects cancel each other out so that pH has no significant effect on the absorption coefficient of the protein solution.

We demonstrated that there were differences in THz absorption of two different types of proteins, which could help distinguish between tubular proteinuria and glomerular proteinuria, the most common type of proteinuria in the clinic. Proteins with a molecular weight less than 20.0 kDa pass the glomerular capillary wall easily (Larson, 1994). Conversely, albumin, with a molecular weight of 65.0 kDa and negative charge, is restricted under normal conditions. The low molecular weight proteins are largely reabsorbed at the proximal tubule, only few amounts are excreted. Thus, tubular proteinuria is dominated by low molecular weight proteins, such as β2-microglobulin (11.7 kDa), and the total amount of the tubular proteins is very small in urine. β2-microglobulin tends to accumulate in the blood and urine of chronic renal failure patients (Winchester et al., 2003). On the other hand, glomerular proteinuria has a large amount of protein and its composition is dominated by albumin. Albuminuria is generally regarded as an excellent marker for assessing early renal damage in diabetes and hypertension (Nah et al., 2017). Distinguishing these two proteins is instructive in diagnosing of proteinuria.

We have initially demonstrated the feasibility of THz in the detection of urinary protein. However, future studies could benefit from the application of data processing algorithms based on the Principal Component Analysis or Karhunen–Loève Transform (KLT) to classify different concentrations of proteinuria (Zaharov et al., 2014; Shao et al., 2022).

## Conclusion

The screening methods of urine samples should clearly separate samples without any indication for renal or genitourinary tract disorders from those samples which need further examination. In our study, a significantly different in absorption coefficient was found between proteinuria and non-proteinuria by THz-TDS method. Further analysis found that urinary protein and solvent water played a key role in this difference. At the same time, we investigated the effect of pH value (6.0–9.0) on urine sample and found that the absorption coefficient of urine sample did not change under different pH value by THz-TDS method. Finally, the absorption spectra of β2-microglobulin and albumin were studied, due to differences in conformation and molecular weight, these two proteins showed significant differences characteristics in absorption coefficient. Our study confirms that THz-TDS method can be used for proteinuria detection with ability to distinguish different proteinuria (renal tubular proteinuria and glomerular proteinuria). Moreover, THz spectroscopy has the potential to overcome the limitations of dipstick method by accurate detection of proteinuria even under alkaline condition. THz-TDS is a label-free method and shows great potential in the field of proteinuria detection. Further studies will establish a complete THz-TDS based system for the quantitative analysis of proteinuria.

## Data Availability

The raw data supporting the conclusions of this article will be made available by the authors, without undue reservation.
